# “Attacks” or “Whistling”: Impact of Questionnaire Wording on Wheeze Prevalence Estimates

**DOI:** 10.1371/journal.pone.0131618

**Published:** 2015-06-26

**Authors:** Anina M. Pescatore, Ben D. Spycher, Caroline S. Beardsmore, Claudia E. Kuehni

**Affiliations:** 1 Institute of Social and Preventive Medicine, University Bern, Bern, Switzerland; 2 Division of Child Health, Department of Infection, Immunity and Inflammation, University of Leicester, Leicester, United Kingdom; Research Center Borstel, GERMANY

## Abstract

**Background:**

Estimates of prevalence of wheeze depend on questionnaires. However, wording of questions may vary between studies. We investigated effects of alternative wording on estimates of prevalence and severity of wheeze, and associations with risk factors.

**Methods:**

White and South Asian children from a population-based cohort (UK) were randomly assigned to two groups and followed up at one, four and six years (1998, 2001, 2003). Parents were asked either if their child ever had “attacks of wheeze” (attack group, N=535), or “wheezing or whistling in the chest” (whistling group, N=2859). All other study aspects were identical, including questions about other respiratory symptoms.

**Results:**

Prevalence of *wheeze ever* was lower in the attack group than in the whistling group for all surveys (32 vs. 40% in white children aged one year, p<0.001). Prevalence of other respiratory symptoms did not differ between groups. Wheeze tended to be more severe in the attack group. The strength of association with risk factors was comparable in the two groups.

**Conclusions:**

The wording of questions on wheeze can affect estimates of prevalence, but has less impact on measured associations with risk factors. Question wording is a potential source of between-study-heterogeneity in meta-analyses.

## Introduction

Questionnaires are the main tool used to estimate prevalence and time trends of wheezing in childhood [1, 2]. They are also widely used to study the natural history of wheeze, to determine risk factors of wheeze and to define wheeze phenotypes [3–8]. In most questionnaire surveys, parents are asked if the child has ever wheezed in life (*wheeze ever*), and if s/he has wheezed during the past 12 months (*current wheeze*). But questionnaires do not always use the same wording to assess wheeze. Some questionnaires used the phrase “attacks of wheeze” [9–12], while others (e.g., those used in the International Study on Asthma and Allergies in Childhood [ISAAC]) refer to wheeze as “wheezing or whistling in the chest” [13]. The first phrase describes wheezing as a discrete episode, while the second phrasing describes a broader range of symptom severity and patterns, and includes children with mild wheeze that does not present as attacks.

Given the breadth of the latter definition, one would expect higher prevalence estimates from surveys that use “wheezing or whistling in the chest” than from those that use “attacks of wheeze”. If phrasing affects prevalence estimates, then results from studies that formulate the question differently might not be comparable, and combining data across studies in meta-analyses may be misleading. To our knowledge, no previous study has tested this hypothesis and quantified the effects of differently worded questions on prevalence estimates of wheeze in preschool and young school children.

Our goal was to determine the effect of the wording of the question used to assess wheeze ever on prevalence estimates of wheeze, indicators of wheeze severity, and its associations with risk factors in a population-based respiratory cohort study.

## Methods

### Study design and study population

We analysed data from a population-based respiratory cohort from Leicestershire, UK, described in detail elsewhere [14]. In brief, the cohort consists of two sub-cohorts, each of which represents a random sample of all children born between 1993 and 1997 in the study area, stratified by ethnicity (white and South Asian). One sub-cohort included children born between May 1993 and April 1997, and was used to study time trends in prevalence of wheeze [15]. The key questions about respiratory symptoms were worded exactly the same in the questionnaires for these children as they had been in questionnaires used in a previous cohort of children, born eight years earlier [14]. The second sub-cohort included children, born between May 1996 and April 1997, who received a similar questionnaire. Most questions were the same as for the first group, but the *wheeze ever* question had the same wording as the ISAAC questionnaire [13].

Parents in both cohorts received respiratory questionnaires in 1998, 2001, 2003, 2006 and 2010. Parents in the second sub-cohort received an additional questionnaire in 1999. Apart from this, study methodology was largely identical for both sub-cohorts including: mailing dates, accompanying letter, questionnaire length and format, and wording of the large majority of questions.

For this study we used data from the 1998, 2001 and 2003 surveys. In order to ensure that the age distribution was comparable, while maximising the size of the two groups, we restricted the analysis to children born May 1^st^ 1996—April 30^th^ 1997 for both sub-cohorts.

### Ethics statement

The Leicestershire Health Authority Research Ethics Committee (Leicester, UK) approved this study.

All information related to individuals from the cohort was made anonymous to investigators prior to analysis.

### Questionnaires

The relevant questionnaire items for this study are published in the supporting information to this paper (S1 Fig).

The question related to *wheeze ever* differed between the sub-cohorts. The sub-cohort used for the study on time trends (referred to as ‘the attack group’) was asked the question: “Has your child ever had **attacks of wheezing**?” The other sub-cohort (referred to as ‘the whistling group’) was asked the question from the ISAAC questionnaire: “Has your child ever had **wheezing or whistling** in the chest at any time in the past?”

Both groups were asked identical questions asking about *current wheeze* and other respiratory symptoms related to the past 12 months, including night cough, chronic rhinitis and ear infections. All these items came from the ISAAC questionnaire [13], except for the question about ear infections. Questions about wheeze severity were also worded identically in both groups, namely activity disturbance, and sleep disturbance due to wheeze, as well as wheeze without colds. The same wording was also used in both groups for most risk factors assessed by questionnaire.

### Statistical analyses

First, we assessed prevalence of *wheeze ever*, *current wheeze*, night cough, chronic rhinitis and any ear infection at ages one, four and six years in both groups. We used logistic regression and estimated odds ratios (ORs) to compare the groups. The questions about *current wheeze* and the other respiratory symptoms, which were worded identically, served as control questions, i.e. any prevalence differences between the sub-cohorts for these questions cannot be due to their wording. We estimated ORs with and without further adjustment for a range of potential risk factors, which may have been distributed unequally by chance between the groups: sex, exact age, breast feeding, nursery care, number of siblings, pre- and postnatal exposure to environmental tobacco smoke (ETS), parental asthma and parental hay fever, Townsend score (an area-based deprivation measure) [16] and parental education.

Second, we assessed prevalence of indicators of wheeze severity, namely activity disturbance and sleep disturbance due to wheeze, and having wheeze without a cold at ages one, four and six years.

Third, we investigated if the strength of association between *wheeze ever* and risk factors differed between groups. For this, we included interaction terms between group membership and the above-mentioned risk factors in multivariable logistic regressions. We used Stata 12.1 (Stata Corporation, Austin, Texas) to analyse the data.

White and South Asian children were analysed separately because these ethnic groups have previously been shown to have different symptoms and triggers of wheeze, lung function, parental understanding of wheeze and wheeze-related health service use [17–21].

## Results

### Study population

A total of 5300 children received a questionnaire in 1998, and were born between May 1^st^ 1996 and April 30^th^ 1997. Of these, 4068 were white children and 1232 were South Asians (S2 Fig). The questionnaire that asked about attacks of wheeze was given to 1077 children. The questionnaire that asked about whistling in the chest was given to 4223 children. Response rates in 1998 were 78.5% (845/1077) for the attack group and 79.1% (3401/4223) for the whistling group. In 2001, response rates were 61.2% (633/1035) for the attack group and 64.7% (2632/4068) for the whistling group. In 2003, response rates were 50.0% (522/1054) for the attack group and 51.4% (2100/4082) for the whistling group. Less than 1.2% of questions about *wheeze ever* were missing for white children, and less than 2.4% of questions about *wheeze ever* were missing for South Asian children in both groups and for all survey years.

The attack group and the whistling group were similar in sex, age, prenatal and perinatal factors, environmental exposures, socioeconomic factors and parental history of atopic disorders (white children Table 1, South Asian children Table A in S1 File).

**Table 1:** Characteristics of the study population at age one year (white children).

	Attack group (N = 534)	Whistling group (N = 2859)	p-value§
**Demographics**			
Male [N, %]	284 (53.2)	1489 (52.1)	0.640
Age (years) [mean, sd]	1.49 (0.29)	1.52 (0.30)	0.025
**Prenatal and perinatal factors**			
Prenatal ETS exposure* [N, %]	121 (23.3)	592 (21.1)	0.275
Gestational age <37 weeks [N, %]	32 (6.0)	188 (6.6)	0.616
Birth weight <2500 g [N, %]	31 (5.8)	167 (5.8)	0.974
**Environmental exposure**			
Breastfed [N, %]	290 (54.5)	1578 (55.6)	0.642
Nursery care [N, %]	142 (26.6)	766 (27.2)	0.786
Older siblings [N, %]	344 (69.4)	1794 (65.5)	0.099
Postnatal ETS exposure [N, %]	198 (37.1)	1114 (39.1)	0.379
**Socioeconomic factors**			
Townsend Deprivation Index†			0.464
more affluent [N, %]	214 (41.1)	1238 (43.8)	
Average [N, %]	194 (37.2)	1027 (36.3)	
more deprived [N, %]	113 (21.7)	563 (19.9)	
High parental education‡ [N, %]	299 (60.2)	1521 (59.2)	0.684
**Parental history of atopy**			
Wheeze/asthma (mother or father) [N, %]	198 (37.3)	1034 (36.9)	0.861
Hay fever (mother or father) [N, %]	263 (52.5)	1363 (51.4)	0.657

ETS: environmental tobacco smoke

*Attack group: "Did she smoke in the year this child was born?"; Whistling group: "Did she smoke during the pregnancy with this child?"

^†^ The categories cover the following Townsend Deprivation Index intervals: more affluent: [-6.222, -1.397]; average: [-1.396, 2.828]; more deprived: [2.829, 11.072]

^‡^ Age at end of education >16 years (mother or father)

^§^ Chi squared tests (except for Age: Wilcoxon rank-sum test)

### Prevalence of wheeze and other respiratory symptoms

At age one year, prevalence of *wheeze ever* among white children was 31.8% in the attack group and 39.6% in the whistling group (p<0.001, Table 2). The absolute difference in prevalence was 7.8%, which corresponds to a 19.7% lower prevalence in the attack group than in the whistling group. The absolute difference in prevalence of *wheeze ever* at age four was 9.7%, and at age six it was 9.0%. Prevalence of *current wheeze* and other respiratory symptoms, which was determined by questions with identical wording, was comparable between the attack group and the whistling group at ages one, four and six (Table 2).

**Table 2:** Prevalence of wheeze and other respiratory symptoms (white children).

**At age one year**	Attack group (N = 534)	Whistling group (N = 2859)		Absolute difference	Relative difference to whistling group
	n %	n %	p-value†	%	%
*Differently worded* *					
Wheeze ever	168 (31.8)	1122 (39.6)	<0.001	-7.8	-19.7
*Identically worded (referring to past 12 months)*:					
Wheeze current	177 (33.7)	1047 (37.4)	0.106	-3.7	-9.9
Night cough	115 (21.7)	647 (22.9)	0.536	-1.2	-5.2
Chronic rhinitis	170 (31.9)	906 (32.0)	0.973	-0.1	-0.3
Any ear infections	234 (46.2)	1239 (44.1)	0.397	2.1	4.8
**At age four years**	N = 412	N = 2266			
	n%	n %	p-value†		
*Differently worded* *					
Wheeze ever	109 (26.7)	816 (36.4)	<0.001	-9.7	-26.6
*Identically worded (referring to past 12 months)*:					
Wheeze current	62 (15.1)	395 (17.6)	0.207	-2.5	-14.2
Night cough	107 (26.4)	604 (27.1)	0.785	-0.7	-2.6
Chronic rhinitis	124 (30.2)	771 (34.4)	0.093	-4.2	-12.2
Any ear infections	142 (35.9)	788 (35.2)	0.772	0.7	2.0
**At age six years**	N = 353	N = 1805			
	n %	n %	p-value†		
*Differently worded* *					
Wheeze ever	95 (27.2)	649 (36.2)	0.001	-9.0	-24.9
*Identically worded (referring to past 12 months)*:					
Wheeze current	47 (13.4)	251 (14.1)	0.752	-0.7	-5.0
Night cough	85 (24.4)	442 (24.7)	0.929	-0.3	-1.2
Chronic rhinitis	100 (28.6)	527 (29.4)	0.753	-0.8	-2.7
Any ear infections	107 (31.8)	545 (31.7)	0.965	0.1	0.3

*A few questionnaire items had different wording in the two groups. Among them was the question about wheeze ever: Attack group: "Has your child ever had attacks of wheezing?"; Whistling group: "Has your child ever had wheezing or whistling in the chest at any time in the past?" Based on this, the groups were labeled "attack group" and "whistling group".

^†^ Chi squared tests

**Fig 1:**
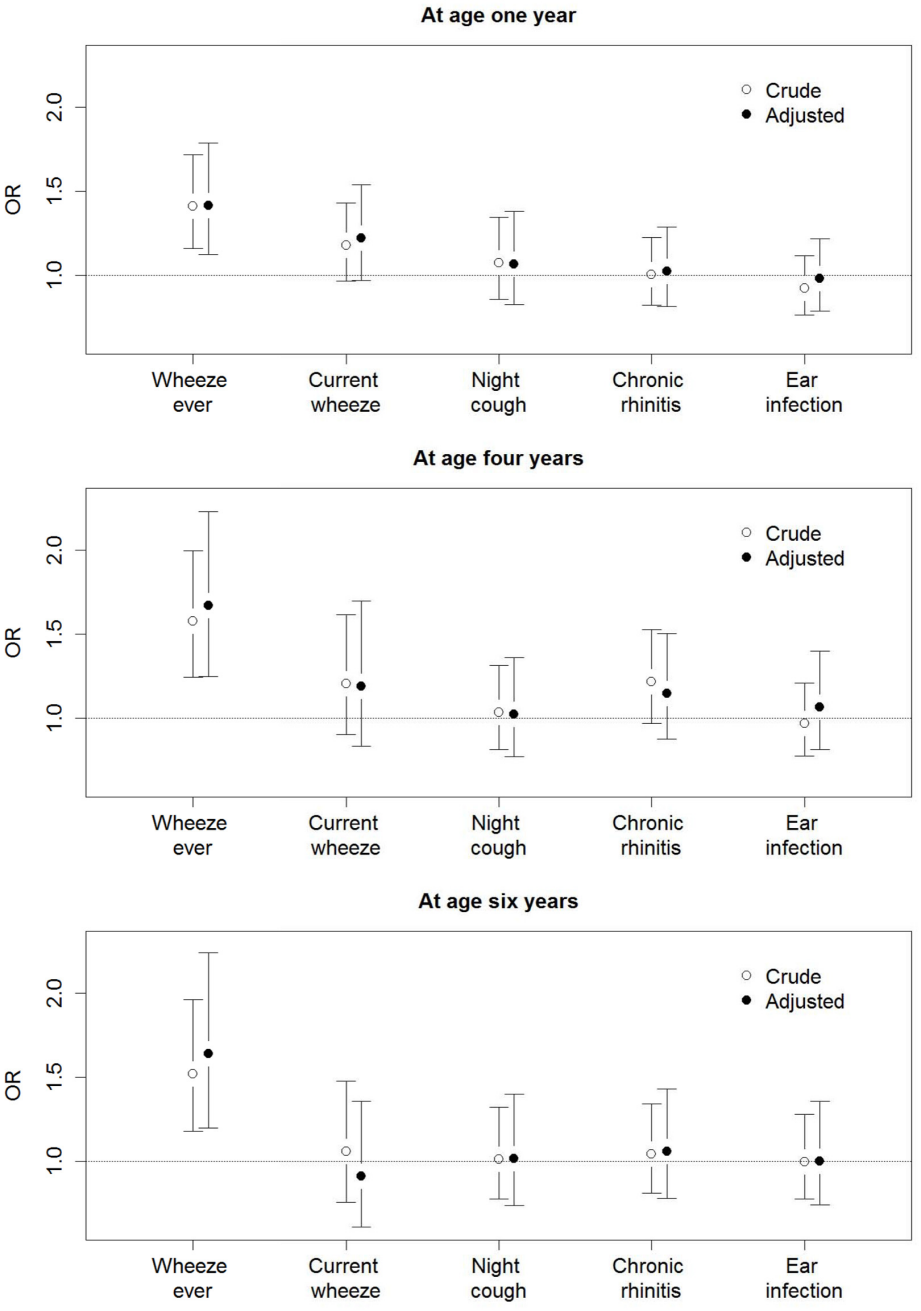
Crude and adjusted odds ratios for respiratory symptoms in white children. The odds ratios compare the whistling group to the attack group (adjusted for sex, exact age, breast feeding, nursery care, number of siblings, pre- and postnatal exposure to environmental tobacco smoke (ETS), parental asthma and parental hay fever, Townsend score (an area-based deprivation measure) and parental education). The error bars denote 95% confidence intervals.

Adjusting for risk factors for wheeze did not affect our findings at any age (Fig 1). At the age of one year, when we compared white children in the whistling group and the attack group, unadjusted OR for *wheeze ever* was 1.41 (95% CI 1.16, 1.72) and adjusted OR was 1.42 (1.12, 1.78). For *current wheeze*, unadjusted OR was 1.18 (0.97, 1.43) and adjusted OR was 1.22 (0.97, 1.54). Other respiratory questions were also similar between groups, with adjusted OR of 1.07 (0.82, 1.38) for night cough, 1.02 (0.81, 1.29) for chronic rhinitis, and 0.98 (0.79, 1.22) for any ear infection.

South Asian children reported less *wheeze ever* than white children (Table B in S1 File). But, as in white children, prevalence of *wheeze ever* also differed between the attack group and the whistling group. There were absolute differences of 6.0% (age one year), 7.5% (age four years) and 11.0% (age six years), while prevalence of *current wheeze* and other respiratory symptoms were more similar at all ages between the two groups. ORs, with and without adjusting for confounders, were comparable (S3 Fig).

### Severity of wheeze

Among white children aged one with wheeze, some indicators of severity of wheeze were more common in the attack group than in the whistling group (activity disturbance [16.6 vs. 9.7%] and sleep disturbance [64.2 vs 54.3%]). A severity difference was still seen at age four, but not at age six (Table 3). In South Asians, reported severity of wheeze varied little between the groups (Table C in S1 File).

**Table 3:** Prevalence of indicators of wheeze severity (white children).

	At age one year			At age four years			At age six years		
	Attack group (N = 412)	Whistling group (N = 2266)		Attack group (N = 412)	Whistling group (N = 2266)		Attack group (N = 353)	Whistling group (N = 1805)	
	N %	N %	p-value†	N %	N %	p-value†	N %	N %	p-value†
*Asked to parents reporting wheeze ever or current in their children (indicators refer to past 12 months)*:									
Activity disturbed due to wheeze (moderately or a lot)	32 (16.6)	111 (9.7)	0.005	18 (16.8)	58 (7.8)	0.002	9 (9.5)	51 (8.4)	0.718
Sleep disturbed due to wheeze	120 (64.2)	613 (54.3)	0.012	45 (42.1)	263 (35.3)	0.174	29 (31.2)	173 (28.1)	0.537
Wheeze without colds	50 (26.0)	367 (32.1)	0.096	33 (30.3)	178 (22.8)	0.084	21 (22.1)	136 (21.4)	0.867

^†^ Chi squared tests

### Association with risk factors

There was little evidence that the strength of association between *wheeze ever* and potential risk factors differed between groups for white or for South Asian children: p-values for interaction tests were >0.1, except for parental asthma and hay fever in children aged one, and sex in children aged six years (whites only) (Table 4 and Tables D and E in S1 File). Adjusted results for the associations of the investigated risk factors with *wheeze ever* were comparable to unadjusted ones (data not shown).

**Table 4:** Association of different risk factors with wheeze ever in white children aged one year (unadjusted).

	Attack group (N = 534)		Whistling group (N = 2859)		
	Odds Ratio (OR)	95% CI	OR	95% CI	p-value for interaction (Risk factor x group)
Male	1.13	(0.78,1.63)	1.43	(1.23,1.67)	0.236
Age (years)	0.97	(0.52,1.81)	0.94	(0.73,1.21)	0.923
Gestational age <37 weeks	1.72	(0.84,3.55)	1.26	(0.94,1.70)	0.438
Birth weight <2500 g	2.26	(1.08,4.74)	1.15	(0.84,1.58)	0.100
Nursery care at age one year	0.93	(0.61,1.42)	1.00	(0.85,1.19)	0.748
Older siblings	1.33	(0.88,2.01)	1.15	(0.98,1.36)	0.539
Parental history of wheeze/asthma (mother or father)	3.16	(2.16,4.62)	1.89	(1.61,2.21)	0.014
Parental hay fever (mother or father)	1.95	(1.32,2.89)	1.34	(1.14,1.57)	0.079
Breastfed	0.70	(0.49,1.02)	0.70	(0.60,0.81)	0.950
Prenatal ETS exposure*	1.35	(0.88,2.07)	1.98	(1.65,2.38)	0.107
Postnatal ETS exposure at age one year	1.85	(1.27,2.69)	1.63	(1.40,1.90)	0.536
Townsend Deprivation Index†					0.344
more affluent (compared to average)	1.22	(0.80,1.87)	1.12	(0.95,1.33)	
more deprived (compared to average)	1.55	(0.95,2.51)	2.09	(1.71,2.57)	
High parental education‡	0.72	(0.49,1.07)	0.71	(0.61,0.84)	0.949

ETS: environmental tobacco smoke

*Attack group: "Did she smoke in the year this child was born?"; Whistling group: "Did she smoke during the pregnancy with this child?"

^†^ The categories cover the following Townsend Deprivation Index intervals: more affluent: [-6.222, -1.397]; average: [-1.396, 2.828]; more deprived: [2.829, 11.072]; We used a likelihood-ratio test to calculate the p-value for interaction for this variable

^‡^ Age at end of education >16 years (mother or father)

## Discussion

Prevalence of *wheeze ever* in preschool and young school children was lower when parents were asked if their child had “attacks of wheezing” than when they were asked if their child had suffered from “wheezing or whistling in the chest”. The absolute difference in prevalence ranged from 8–10% in different age groups of white children. Among those who reported wheeze, severity of wheeze tended to be higher in the attack group than in the whistling group, particularly in one to two year-olds. In contrast, the strength of the association of *wheeze ever* with risk factors was less affected by the wording.

To our knowledge, this is the first study to systematically evaluate the way wording used to assess *wheeze ever* affects estimates of wheeze prevalence in preschool and young school children. In a Norwegian study, adults aged 15–70 years filled in two different respiratory questionnaires at an interval of about two hours. The authors found that prevalence of chest wheeze varied from 18.7% to 24.5%, depending on the wording of the question [22]. Ekerljung et al distributed booklets, each containing two respiratory questionnaires, to 16–75 year-old subjects in Sweden [23]. They observed that different wording of questions related to respiratory symptoms resulted in a difference in prevalence estimates of up to 8.8%, and that differences in question layout resulted in a difference up to 6.6%. Our results agree with findings of Patel et al, who reviewed global variations in wheeze prevalence in schoolchildren [24] and found that ISAAC studies reported significantly higher prevalence than non-ISAAC studies. However, their report did not account for regional variation of wheeze prevalence within countries [25–27], and the studies they compared were different in many methodological aspects, which made it difficult to attribute their findings specifically to wording.

Our study included two fully comparable groups that differed only in the wording of the questionnaire they received. All children were randomly sampled at the same time, from the same sampling frame, and were simultaneously surveyed using otherwise identical methods. Thus our study setting was comparable to a randomized trial. Questions on *current wheeze* and other respiratory symptoms like night cough, rhinitis and ear infections were identically worded and could therefore be used as control questions. As expected, their prevalence was similar in both groups, and confirmed the comparability of the two groups regarding general respiratory morbidity.

We included a substantial number of South Asians in the study, a significant ethnic minority group in the UK. Findings were similar in both ethnic groups, suggesting that our results might generalize to non-white ethnic groups.

We based our study on English language questionnaires administered to preschool and young school children in the UK. We do not yet know how wording affects prevalence of wheeze in other languages or countries, or in older children.

Our results suggest that parents are more reluctant to confirm wheeze ever’ when the question includes the word “attack”. Observed differences persisted throughout early childhood. When wheeze prevalence estimates are compared, or when data are pooled in meta-analyses, it is therefore important to report whether or not questions were identical, and to consider the wording of questions to be a potential source of heterogeneity. Furthermore, the wording of questions should be carefully considered when children are classified into wheezing phenotypes. Reported severity of wheeze tended to be higher in children with “attacks” of wheeze. This supports our initial assumption that fewer children would be reported to have wheezing “attacks”, and would, on average, report more severe symptoms than children reported to have “wheezing or whistling in the chest”.

The strength of associations of *wheeze ever* with different risk factors was comparable between groups among white and South Asians children, at any age. Results went in the same direction and were of comparable effect size. Exceptions were parental asthma for white children aged one year, and sex of children aged six years, though these might have occurred by chance, given multiple testing. This suggests that wording is less likely to affect studies that investigate associations of wheeze with risk factors.

In summary, we found that the way the question on wheeze is worded can considerably affect estimates of prevalence of wheeze in young children. Wording had less impact on estimated strength of association with risk factors. The wording of the questionnaire must be considered as a potential source of between-study-heterogeneity in meta-analyses.

## Supporting Information

S1 Fig Original questions about wheeze and other respiratory symptoms. The questions were sent to the attack group and the whistling group in 1998, 2001 and 2003 in Leicestershire, UK.
(TIFF)

S2 Fig Response rate of study population for questionnaire surveys in 1998, 2001, and 2003. At each survey, the attack group received the question, “Has your child ever had attacks of wheezing?” At each survey, the whistling group received the question. “Has your child ever had wheezing or whistling in the chest at any time in the past?”
(TIF)

S3 Fig Crude and adjusted odds ratios for respiratory symptoms in South Asian children. The odds ratios compare the whistling group to the attack group (adjusted for sex, exact age, breast feeding, nursery care, number of siblings, pre- and postnatal exposure to environmental tobacco smoke (ETS), parental asthma and parental hay fever, Townsend score (an area-based deprivation measure) and parental education). The error bars denote 95% confidence intervals.
(TIF)

S1 File Table A in S1 File. Characteristics of the study population at age 1 year (South Asians). Table B in S1 File. Prevalence of wheeze and other respiratory symptoms (South Asian children). Table C in S1 File. Prevalence of indicators of wheeze severity (South Asian children). Table D in S1 File. Association of different risk factors with *wheeze ever* in white children aged 4 and 6 years (unadjusted). Table E in S1 File. Association of different risk factors with *wheeze ever* in South Asian children aged 1, 4 and 6 years (unadjusted)
(DOCX)
