# Supplementary figures and images for: “Attacks” or “Whistling”: Impact of Questionnaire Wording on Wheeze Prevalence Estimates
Source: PLoS One. 2015 Jun 26;10(6):e0131618. doi: 10.1371/journal.pone.0131618 (PMC4482591; doi:10.1371/journal.pone.0131618)

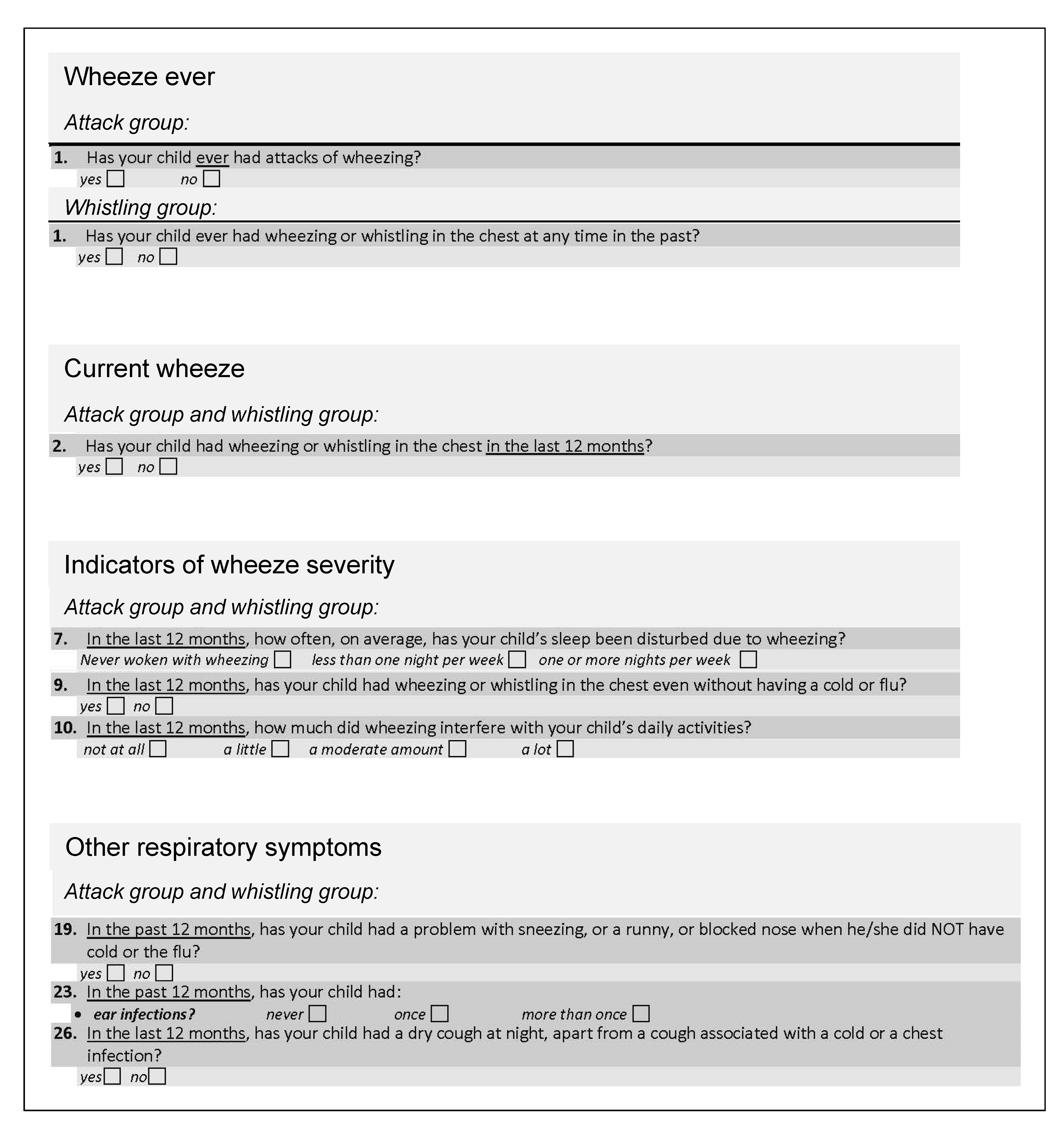

Supplement: S1 Fig — The questions were sent to the attack group and the whistling group in 1998, 2001 and 2003 in Leicestershire, UK. (TIFF) [file pone.0131618.s001.tiff]

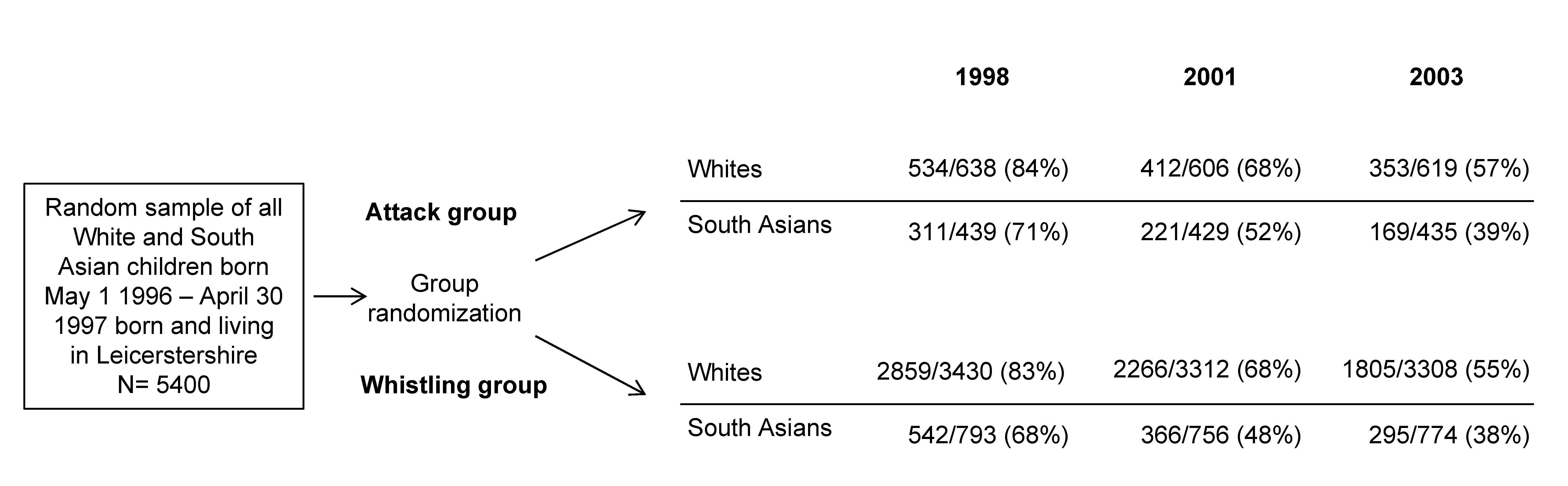

Supplement: S2 Fig — At each survey, the attack group received the question, “Has your child ever had attacks of wheezing?” At each survey, the whistling group received the question. “Has your child ever had wheezing or whistling in the chest at any time in the past?” (TIF) [file pone.0131618.s002.tif]

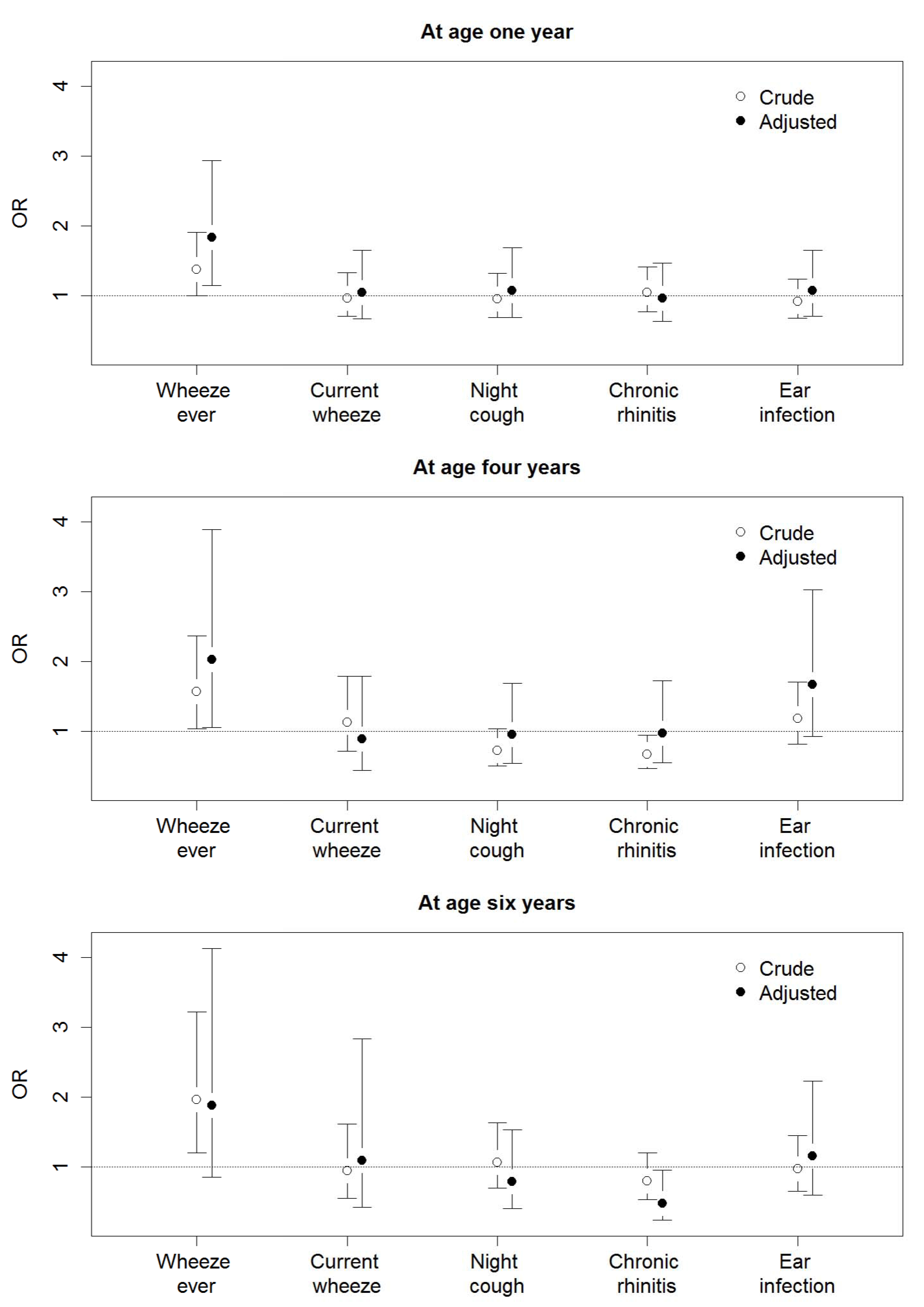

Supplement: S3 Fig — The odds ratios compare the whistling group to the attack group (adjusted for sex, exact age, breast feeding, nursery care, number of siblings, pre- and postnatal exposure to environmental tobacco smoke (ETS), parental asthma and parental hay fever, Townsend score (an area-based deprivation measure) and parental education). The error bars denote 95% confidence intervals. (TIF) [file pone.0131618.s003.tif]
