# Supplementary material for: “Attacks” or “Whistling”: Impact of Questionnaire Wording on Wheeze Prevalence Estimates
Source: PLoS One. 2015 Jun 26;10(6):e0131618. doi: 10.1371/journal.pone.0131618 (PMC4482591; doi:10.1371/journal.pone.0131618)
Supplement: S1 File — Table A in S1 File. Characteristics of the study population at age 1 year (South Asians). Table B in S1 File. Prevalence of wheeze and other respiratory symptoms (South Asian children). Table C in S1 File. Prevalence of indicators of wheeze severity (South Asian children). Table D in S1 File. Association of different risk factors with wheeze ever in white children aged 4 and 6 years (unadjusted). Table E in S1 File. Association of different risk factors with wheeze ever in South Asian children aged 1, 4 and 6 years (unadjusted) (DOCX) [file pone.0131618.s004.docx]

# “Attacks” or “whistling”: impact of questionnaire wording on wheeze prevalence estimates

Anina M. Pescatore^1^, Ben D. Spycher^1^, Caroline S. Beardsmore^2^, Claudia E. Kuehni^1*^

^1^Institute of Social and Preventive Medicine, University Bern, Bern, Switzerland

^2^Division of Child Health, Department of Infection, Immunity and Inflammation, University of Leicester, Leicester, UK

*Corresponding author

Claudia Kuehni

E-mail: kuehni@ispm.unibe.ch (CEK)

# Supporting Information

**Number of tables and figures**

5 tables, 3 figures

# Tables

| **Table A in S1 File. Characteristics of the study population at age 1 year (South Asians)** | | | | | |
| --- | --- | --- | --- | --- | --- |
|  | Attack group (N=311) | | Whistling group (N=542) | |  |
|  | N | % | N | % | p-value§ |
| **Demographics** |  |  |  |  |  |
| Male | 160 | (51.4) | 286 | (52.8) | 0.710 |
| Age (months) [mean, sd] | 1.57 | (0.30) | 1.57 | (0.31) | 0.710 |
| **Prenatal and perinatal factors** |  |  |  |  |  |
| Prenatal ETS exposure* | 2 | (0.7) | 6 | (1.2) | 0.508 |
| Gestational age <37 weeks | 18 | (6.5) | 45 | (11.3) | 0.033 |
| Birth weight <2500 g | 28 | (10.1) | 60 | (15.1) | 0.057 |
| **Environmental exposure** |  |  |  |  |  |
| Breastfed | 225 | (73.1) | 391 | (72.9) | 0.974 |
| Nursery care | 60 | (19.4) | 87 | (16.4) | 0.279 |
| Older siblings | 213 | (74.0) | 358 | (69.6) | 0.196 |
| Postnatal ETS exposure | 82 | (26.5) | 147 | (27.1) | 0.832 |
| **Socioeconomic factors** |  |  |  |  |  |
| Townsend DeprivationIndex† |  |  |  |  | 0.085 |
| more affluent | 45 | (14.7) | 108 | (20.0) |  |
| average | 99 | (32.2) | 146 | (27.0) |  |
| more deprived | 163 | (53.1) | 286 | (53.0) |  |
| High parental education‡ | 218 | (85.8) | 392 | (84.1) | 0.543 |
| **Parental history of atopy** |  |  |  |  |  |
| Wheeze/asthma (mother or father) | 62 | (20.1) | 115 | (21.8) | 0.573 |
| Hay fever (mother or father) | 102 | (34.7) | 176 | (34.9) | 0.964 |
| ETS: environmental tobacco smoke | | | | | |
| *Attack group: "Did she smoke in the year this child was born?"; Whistling group: "Did she smoke during the pregnancy with this child?" | | | | | |
| †The categories cover the following Townsend Deprivation Index intervals: | | | | | |
| more affluent: [-6.222, -1.397]; average: [-1.396, 2.828]; more deprived: [2.829, 11.072] | | | | | |
| ‡ Age at end of education >16 years (mother or father) | | | | | |
| §Chi squared tests (except for Age : Wilcoxon rank-sum test) | | | | | |

| **Table B in S1 File. Prevalence of wheeze and other respiratory symptoms (South Asian children)** | | | | | | | |
| --- | --- | --- | --- | --- | --- | --- | --- |
| **At age 1 year** | Attack group (N=311) | | Whistling group (N=542) | |  | Absolute difference | Relative difference to whistling group |
|  | n | % | n | % | p-value† | % | % |
| *Differently worded** |  |  |  |  |  |  |  |
| Wheeze ever | 70 | (22.7) | 155 | (28.7) | 0.055 | -6.0 | -20.9 |
| *Identically worded (referring to past 12 months):* | | | | | |  |  |
| Wheeze current | 81 | (26.7) | 140 | (26.0) | 0.810 | 0.7 | 2.7 |
| Night cough | 77 | (25.2) | 128 | (24.2) | 0.754 | 1.0 | 4.1 |
| Chronic rhinitis | 95 | (31.1) | 173 | (32.0) | 0.790 | -0.9 | -2.8 |
| Any ear infections | 105 | (36.7) | 186 | (34.7) | 0.566 | 2.0 | 5.8 |
| **At age 4 years** | N=221 | | N=366 | |  |  |  |
|  | n | % | n | % | p-value† |  |  |
| *Differently worded** |  |  |  |  |  |  |  |
| Wheeze ever | 40 | (18.4) | 95 | (26.1) | 0.034 | -7.7 | -29.5 |
| *Identically worded (referring to past 12 months):* | | | | | |  |  |
| Wheeze current | 33 | (15.2) | 61 | (16.8) | 0.614 | -1.6 | -9.5 |
| Night cough | 77 | (36.0) | 102 | (28.7) | 0.071 | 7.3 | 25.4 |
| Chronic rhinitis | 90 | (41.9) | 117 | (32.3) | 0.021 | 9.6 | 29.7 |
| Any ear infections | 63 | (31.3) | 126 | (35.0) | 0.380 | -3.7 | -10.6 |
| **At age 6 years** | N=169 | | N=295 | |  |  |  |
|  | n | % | n | % | p-value† |  |  |
| *Differently worded** |  |  |  |  |  |  |  |
| Wheeze ever | 26 | (15.7) | 77 | (26.7) | 0.007 | -11.0 | -41.2 |
| *Identically worded (referring to past 12 months):* | | | | | |  |  |
| Wheeze current | 25 | (15.4) | 42 | (14.6) | 0.820 | 0.8 | 5.5 |
| Night cough | 46 | (28.0) | 85 | (29.3) | 0.776 | -1.3 | -4.4 |
| Chronic rhinitis | 58 | (34.9) | 87 | (30.0) | 0.276 | 4.9 | 16.3 |
| Any ear infections | 60 | (39.0) | 103 | (38.1) | 0.869 | 0.9 | 2.4 |
| *A few questionnaire items had different wording in the two groups. Among them was the question about *wheeze ever*: Attack group: "Has your child ever had attacks of wheezing?" Whistling group: "Has your child ever had wheezing or whistling in the chest at any time in the past?" Based on this, the groups were labeled "attack group" and "whistling group". | | | | | | | |
| †Chi squared tests | | | | | | | |

| **Table C in S1 File. Prevalence of indicators of wheeze severity (South Asian children)** | | | | | | | | | | | | | | | |
| --- | --- | --- | --- | --- | --- | --- | --- | --- | --- | --- | --- | --- | --- | --- | --- |
|  | **At age 1 year** | | | | | **At age 4 years** | | | | | **At age 6 years** | | | | |
|  | Attack group (N=311) | | Whistling group (N=542) | |  | Attack group (N=221) | | Whistling group (N=366) | |  | Attack group (N=169) | | Whistling group (N=295) | |  |
|  | N | % | N | % | p-value† | N | % | N | % | p-value† | N | % | N | % | p-value† |
| *Asked to parents reporting wheeze ever or current in their children (indicators refer to past 12 months):* | | | | | | | | | | | | |  |  |  |
| Activity disturbed due to wheeze (moderately or a lot) | 18 | (17.3) | 25 | (14.0) | 0.450 | 9 | (15.8) | 16 | (15.0) | 0.887 | 6 | (13.6) | 11 | (12.5) | 0.854 |
| Sleep disturbed due to wheeze | 50 | (49.5) | 86 | (50.6) | 0.863 | 24 | (45.3) | 56 | (53.8) | 0.310 | 16 | (39.0) | 35 | (40.2) | 0.897 |
| Wheeze without colds | 17 | (16.2) | 42 | (22.8) | 0.178 | 6 | (10.3) | 25 | (21.7) | 0.065 | 11 | (23.4) | 21 | (21.9) | 0.837 |
| †Chi squared tests | | | | | | | | | | | | | | | |

| **Table D in S1 File. Association of different risk factors with *wheeze ever* in white children aged 4 and 6 years (unadjusted)** | | | | | |
| --- | --- | --- | --- | --- | --- |
|  | Attack group (N=412) | | Whistling group (N=2266) | |  |
| **At age 4 years** | Odds Ratio (OR) | 95% CI | OR | 95% CI | p-value for interaction (Risk factor x group) |
| Male | 1.12 | (0.72,1.73) | 1.46 | (1.23,1.74) | 0.262 |
| Age (years) | 1.34 | (0.65,2.80) | 1.06 | (0.80,1.40) | 0.546 |
| Gestational age <37 weeks | 1.74 | (0.70,4.33) | 1.35 | (0.94,1.92) | 0.607 |
| Birth weight <2500 g | 3.26 | (1.34,7.90) | 1.37 | (0.94,1.98) | 0.076 |
| Nursery care at age 1 year | 0.92 | (0.55,1.55) | 1.12 | (0.92,1.37) | 0.492 |
| Older siblings | 1.13 | (0.68,1.89) | 1.03 | (0.85,1.25) | 0.744 |
| Parental history of wheeze/asthma (mother or father) | 1.61 | (1.00,2.60) | 2.28 | (1.89,2.75) | 0.188 |
| Parental hay fever (mother or father) | 1.87 | (1.14,3.05) | 1.51 | (1.25,1.83) | 0.430 |
| Breastfed | 0.59 | (0.37,0.94) | 0.77 | (0.64,0.92) | 0.303 |
| Prenatal ETS exposure* | 1.05 | (0.58,1.89) | 1.42 | (1.12,1.81) | 0.352 |
| Postnatal ETS exposure at age 1 year | 1.61 | (1.00,2.58) | 1.36 | (1.12,1.64) | 0.524 |
| Townsend Deprivation Index† |  |  |  |  | 0.937 |
| more affluent (compared to average) | 0.93 | (0.56,1.55) | 0.94 | (0.77,1.14) |  |
| more deprived (compared to average) | 1.48 | (0.83,2.64) | 1.65 | (1.30,2.08) |  |
| High parental education‡ | 0.72 | (0.45,1.16) | 0.86 | (0.71,1.04) | 0.507 |
|  |  |  |  |  |  |
|  | Attack group (N=353) | | Whistling group (N=1805) | |  |
| **At age 6years** | OR | 95% CI | OR | 95% CI |  |
| Male | 0.58 | (0.36,0.93) | 1.44 | (1.19,1.75) | <0.001 |
| Age (years) | 0.71 | (0.32,1.58) | 1.13 | (0.82,1.55) | 0.293 |
| Gestational age <37 weeks | 1.57 | (0.64,3.87) | 1.53 | (1.03,2.27) | 0.954 |
| Birth weight <2500 g | 4.32 | (1.78,10.47) | 1.28 | (0.85,1.92) | 0.014 |
| Nursery care at age 1 year | 0.81 | (0.46,1.41) | 1.03 | (0.83,1.29) | 0.416 |
| Older siblings | 1.20 | (0.70,2.05) | 1.06 | (0.85,1.31) | 0.673 |
| Parental history of wheeze/asthma (mother or father) | 2.28 | (1.38,3.77) | 2.16 | (1.75,2.66) | 0.843 |
| Parental hay fever (mother or father) | 2.27 | (1.33,3.87) | 1.61 | (1.30,1.98) | 0.237 |
| Breastfed | 0.53 | (0.32,0.87) | 0.75 | (0.61,0.93) | 0.195 |
| Prenatal ETS exposure* | 1.46 | (0.78,2.75) | 1.35 | (1.03,1.78) | 0.823 |
| Postnatal ETS exposure at age 1 year | 1.54 | (0.92,2.59) | 1.52 | (1.23,1.89) | 0.962 |
| Townsend Deprivation Index† |  |  |  |  | 0.964 |
| more affluent (compared to average) | 1.00 | (0.58,1.72) | 0.96 | (0.78,1.19) |  |
| more deprived (compared to average) | 1.36 | (0.72,2.56) | 1.44 | (1.10,1.90) |  |
| High parental education‡ | 0.67 | (0.40,1.12) | 0.76 | (0.61,0.94) | 0.665 |
| ETS: environmental tobacco smoke | | | | | |
| *Attack group: "Did she smoke in the year this child was born?"; Whistling group: "Did she smoke during the pregnancy with this child?" | | | | | |
| †The categories cover the following Townsend Deprivation Index intervals: | | | | | |
| more affluent: [-6.222, -1.397]; average: [-1.396, 2.828]; more deprived: [2.829, 11.072] | | | | | |
| We used a likelihood-ratio test to calculate the p-value for interaction for this variable | | | | | |
| ‡Age at end of education >16 years (mother or father) | | | | | |

| **Table E in S1 File. Association of different risk factors with *wheeze ever* in South Asian children aged 1, 4 and 6 years (unadjusted)** | | | | | |
| --- | --- | --- | --- | --- | --- |
|  | Attack group (N=311) | | Whistling group (N=542) | |  |
| **At age 1 year** | Odds Ratio (OR) | 95% CI | OR | 95% CI | p-value for interaction (Risk factor x group) |
| Male | 1.08 | (0.63,1.83) | 0.89 | (0.62,1.30) | 0.578 |
| Age (years) | 0.83 | (0.34,1.99) | 1.05 | (0.57,1.91) | 0.662 |
| Gestational age <37 weeks | 1.01 | (0.32,3.18) | 2.21 | (1.18,4.16) | 0.239 |
| Birth weight <2500 g | 1.20 | (0.48,2.97) | 1.66 | (0.94,2.92) | 0.552 |
| Nursery care at age 1 year | 1.70 | (0.90,3.21) | 1.46 | (0.90,2.38) | 0.709 |
| Older siblings | 1.03 | (0.55,1.94) | 1.51 | (0.98,2.33) | 0.332 |
| Parental history of wheeze/asthma (mother or father) | 1.76 | (0.94,3.29) | 2.13 | (1.38,3.29) | 0.626 |
| Parental hay fever (mother or father) | 1.63 | (0.93,2.85) | 1.71 | (1.15,2.54) | 0.895 |
| Breastfed | 1.48 | (0.79,2.81) | 1.04 | (0.68,1.58) | 0.359 |
| Prenatal ETS exposure* | 3.34 | (0.21,54.17) | 1.25 | (0.23,6.92) | 0.556 |
| Postnatal ETS exposure at age 1 year | 1.16 | (0.64,2.10) | 1.19 | (0.79,1.79) | 0.952 |
| Townsend Deprivation Index† |  |  |  |  | 0.486 |
| more affluent (compared to average) | 1.11 | (0.45,2.77) | 1.22 | (0.00,2.13) |  |
| more deprived (compared to average) | 1.67 | (0.72,3.87) | 1.19 | (0.70,1.96) |  |
| High parental education‡ | 0.67 | (0.30,1.48) | 1.16 | (0.66,2.04) | 0.270 |
|  |  |  |  |  |  |
|  | Attack group (N=221) | | Whistling group (N=366) | |  |
| **At age 4 years** | OR | 95% CI | OR | 95% CI |  |
| Male | 1.64 | (0.82,3.28) | 1.06 | (0.66,1.69) | 0.303 |
| Age (years) | 0.34 | (0.11,1.06) | 0.61 | (0.28,1.29) | 0.412 |
| Gestational age <37 weeks | 1.09 | (0.29,4.07) | 1.18 | (0.49,2.84) | 0.925 |
| Birth weight <2500 g | 0.93 | (0.30,2.93) | 0.66 | (0.29,1.52) | 0.632 |
| Nursery care at age 1 year | 0.64 | (0.25,1.66) | 0.88 | (0.44,1.75) | 0.598 |
| Older siblings | 1.19 | (0.50,2.85) | 0.94 | (0.53,1.66) | 0.656 |
| Parental history of wheeze/asthma (mother or father) | 2.36 | (1.05,5.30) | 2.31 | (1.26,4.26) | 0.968 |
| Parental hay fever (mother or father) | 0.96 | (0.45,2.03) | 1.53 | (0.88,2.66) | 0.320 |
| Breastfed | 0.96 | (0.43,2.15) | 1.11 | (0.60,2.04) | 0.776 |
| Postnatal ETS exposure at age 1 year | 1.85 | (0.86,4.00) | 1.04 | (0.58,1.87) | 0.244 |
| Townsend Deprivation Index† |  |  |  |  | 0.602 |
| more affluent (compared to average) | 0.93 | (0.30,2.92) | 1.51 | (0.76,2.98) |  |
| more deprived (compared to average) | 1.27 | (0.44,3.69) | 1.31 | (0.70,2.46) |  |
|  | 0.80 | (0.27,2.38) | 0.64 | (0.30,1.37) | 0.737 |
|  |  |  |  |  |  |
|  | Attack group (N=169) | | Whistling group (N=295) | |  |
| **At age 6 years** | OR | 95% CI | OR | 95% CI |  |
| Male | 1.33 | (0.57,3.09) | 1.00 | (0.59,1.69) | 0.581 |
| Age (years) | 0.32 | (0.07,1.40) | 0.91 | (0.39,2.15) | 0.231 |
| Gestational age <37 weeks | 3.16 | (0.73,13.71) | 1.70 | (0.72,3.99) | 0.473 |
| Birth weight <2500 g | 1.30 | (0.26,6.46) | 0.78 | (0.33,1.84) | 0.580 |
| Nursery care at age 1 year | 0.58 | (0.16,2.09) | 0.39 | (0.14,1.04) | 0.631 |
| Older siblings | 0.77 | (0.27,2.17) | 0.94 | (0.50,1.74) | 0.752 |
| Parental history of wheeze/asthma (mother or father) | 3.42 | (1.26,9.28) | 2.74 | (1.42,5.29) | 0.717 |
| Parental hay fever (mother or father) | 3.27 | (1.29,8.30) | 1.70 | (0.94,3.10) | 0.248 |
| Breastfed | 3.32 | (0.73,14.97) | 0.78 | (0.41,1.50) | 0.084 |
| Postnatal ETS exposure at age 1 year | 0.65 | (0.20,2.04) | 0.55 | (0.26,1.17) | 0.826 |
| Townsend Deprivation Index† |  |  |  |  | 0.481 |
| more affluent (compared to average) | 0.59 | (0.17,2.04) | 1.07 | (0.52,2.23) |  |
| more deprived (compared to average) | 0.36 | (0.11,1.21) | 0.87 | (0.43,1.73) |  |
|  | 2.03 | (0.25,16.79) | 1.69 | (0.61,4.68) | 0.875 |
| ETS: environmental tobacco smoke | | | | | |
| *Attack group: "Did she smoke in the year this child was born?"; Whistling group: "Did she smoke during the pregnancy with this child?" (dropped at ages 4 and 6 years because there were too few positive answers in the groups at these ages) | | | | | |
| †The categories cover the following Townsend Deprivation Index intervals: | | | | | |
| more affluent: [-6.222, -1.397]; average: [-1.396, 2.828]; more deprived: [2.829, 11.072] | | | | | |
| We used a likelihood-ratio test to calculate the p-value for interaction for this variable | | | | | |
| ‡Age at end of education >16 years (mother or father) | | | | | |

# Figure Legends

**S1 Figure. Original questions about wheeze and other respiratory symptoms.** The questions were sent to the attack group and the whistling group in 1998, 2001 and 2003 in Leicestershire, UK.

**S2 Figure**. **Response rate of study population for questionnaire surveys in 1998, 2001, and 2003.** At each survey, the attack group received the question, “Has your child ever had attacks of wheezing?” At each survey, the whistling group received the question. “Has your child ever had wheezing or whistling in the chest at any time in the past?”

**S3 Figure. Crude and adjusted odds ratios for respiratory symptoms in South Asian children.** The odds ratios compare the whistling group to the attack group (adjusted for sex, exact age, breast feeding, nursery care, number of siblings, pre- and postnatal exposure to environmental tobacco smoke (ETS), parental asthma and parental hay fever, Townsend score (an area-based deprivation measure) and parental education). The error bars denote 95% confidence intervals.
